# The *kdr*-bearing haplotype and susceptibility to *Plasmodium falciparum* in *Anopheles gambiae*: genetic correlation and functional testing

**DOI:** 10.1186/s12936-015-0924-8

**Published:** 2015-10-06

**Authors:** Christian Mitri, Kyriacos Markianos, Wamdaogo M. Guelbeogo, Emmanuel Bischoff, Awa Gneme, Karin Eiglmeier, Inge Holm, N’Fale Sagnon, Kenneth D. Vernick, Michelle M. Riehle

**Affiliations:** Unit of Insect Vector Genetics and Genomics, Department of Parasites and Insect Vectors, CNRS Unit of Hosts, Vectors and Pathogens (URA3012), Lab GGIV, Institut Pasteur, 28 rue du Dr Roux, 75015 Paris, France; Program in Genomics, Boston Children’s Hospital, Harvard Medical School, Boston, MA 02115 USA; Centre National de Recherche et de Formation sur le Paludisme, 01 BP 2208, Ouagadougou, Burkina Faso; Department of Microbiology, University of Minnesota, Saint Paul, MN 55108 USA

**Keywords:** Mosquito, Malaria, Host-pathogen interaction, Haplotype

## Abstract

**Background:**

Members of the *Anophelesgambiae* species complex are primary vectors of human malaria in Africa. It is known that a large haplotype shared between *An. gambiae* and *Anophelescoluzzii* by introgression carries point mutations of the voltage-gated sodium channel gene *para,* including the L1014F *kdr* mutation associated with insensitivity to pyrethroid insecticides. Carriage of L1014F *kdr* is also correlated with higher susceptibility to infection with *Plasmodium falciparum*. However, the genetic mechanism and causative gene(s) underlying the parasite susceptibility phenotype are not known.

**Methods:**

Mosquitoes from the wild Burkina Faso population were challenged by feeding on natural *P. falciparum* gametocytes. Oocyst infection phenotypes were determined and were tested for association with SNP genotypes. Candidate genes in the detected locus were prioritized and RNAi-mediated gene silencing was used to functionally test for gene effects on *P. falciparum* susceptibility.

**Results:**

A genetic locus, Pfin6, was identified that influences infection levels of *P. falciparum* in mosquitoes. The locus segregates as a ~3 Mb haplotype carrying 65 predicted genes including the *para* gene. The haplotype carrying the *kdr* allele of *para* is linked to increased parasite infection prevalence, but many single nucleotide polymorphisms on the haplotype are also equally linked to the infection phenotype. Candidate genes in the haplotype were prioritized and functionally tested. Silencing of *para* did not influence *P. falciparum* infection, while silencing of a predicted immune gene, serine protease *ClipC9,* allowed development of significantly increased parasite numbers.

**Conclusions:**

Genetic variation influencing *Plasmodium* infection in wild *Anopheles* is linked to a natural ~3 megabase haplotype on chromosome 2L that carries the *kdr* allele of the *para* gene. Evidence suggests that *para* gene function does not directly influence parasite susceptibility, and the association of *kdr* with infection may be due to tight linkage of *kdr* with other gene(s) on the haplotype. Further work will be required to determine if ClipC9 influences the outcome of *P. falciparum* infection in nature, as well as to confirm the absence of a direct influence by *para*.

**Electronic supplementary material:**

The online version of this article (doi:10.1186/s12936-015-0924-8) contains supplementary material, which is available to authorized users.

## Background

Throughout sub-Saharan Africa, members of the *Anopheles gambiae* species complex are primary vectors of the human malaria parasite *Plasmodium falciparum*, which is responsible for extensive morbidity and mortality. Phenotypic differences for traits that influence malaria transmission, such as behaviour, *Plasmodium* susceptibility and insecticide resistance can be influenced by genetic variation [1–4].

Mosquito susceptibility to *P. falciparum* is variable and has a strong genetic component, as shown by population-based mapping of quantitative trait loci [3, 5, 6], laboratory-based phenotypic selection of resistant lines [7], and genetic association studies [8, 9]. However, despite a genetic basis for variation in susceptibility to infection with *P. falciparum*, underlying causative single nucleotide polymorphisms (SNP) have neither been identified nor replicated in natural populations.

Similarly, a number of genetic mechanisms influence susceptibility to insecticides [10]. However, unlike the case of parasite susceptibility, individual SNPs have been robustly implicated. Increased resistance to pyrethroid insecticides is influenced in part by a SNP of the voltage-gated sodium channel gene *para* (also called VSG). The mutation of amino acid residue 1014 of the conserved S6 transmembrane segment of para domain II from leucine to phenylalanine (L1014F) or serine (L1014S) is correlated with decreased excitability of the insect nervous system, which is thought to be related to phenotypic insensitivity to insecticide. The allele is named knock-down resistance (*kdr*), and has arisen independently at the homologous position in *An. gambiae* [2] and other insects [11, 12]. Other coding variants in and near *para* can also contribute to pyrethroid-insensitivity [10, 13]. In *An. gambiae*, L1014F is referred to as *kdr*-W because it was originally described in West Africa, whilst L1014S, first described in East Africa, is called *kdr*-E [14, 15]. The geographic labels however, are now misnomers as these resistance loci are not geographically restricted, because L1014F co-occurs with L1014S in East Africa [16] and rates of L1014S have been increasing in West Africa [17, 18].

Mosquito sister taxa *Anopheles coluzzii* and *An. gambiae*, the former M and S molecular forms, respectively [19], display different frequencies of the L1014F mutation. The L1014F allele is nearly fixed in *An. gambiae* in West Africa but segregates in many populations of *An. coluzzii* as a consequence of introgression from *An. gambiae* to *An. coluzzii* occurring within the past 10–15 years [18, 20–23]. Genotype analysis indicates that the introgression of *kdr* L1014F from *An. gambiae* to *An. coluzzii* occurred upon an extended chromosome haplotype [24, 25].

Recent studies have reported a correlation between *P. falciparum* susceptibility and the *kdr* L1014F allele [26, 27]. However, *para* is just one among many genes carried on an extended haplotype, and consequently the *kdr* L1014F SNP variant is a marker not only for propensity to pyrethroid resistance, but equally tags a block of up to 65 linked genes. There are no reports yet on which genes within this haplotype influence parasite susceptibility, or whether particular genes, for example *para*, might display both phenotypes both for parasite and insecticide resistance. The power to genetically dissect the phenotypes by fine-mapping will likely be diminished by the expected low frequency of recombination due to the haplotype structure, which will be further decreased by the centromeric location.

Here, significant genetic association of the *para*-bearing haplotype with susceptibility to wild *P. falciparum* was detected. Because of the limited power of recombinational fine-mapping to distinguish gene phenotypes within the locus, a candidate gene approach was used. Genes in the locus were prioritized by bio-informatic prediction of function. Two chosen candidates were silenced by RNAi and challenged with *P. falciparum*. A candidate gene with strong immune functional prediction, *ClipC9*, influenced parasite infection, while silencing of *para* did not change parasite infection levels. As with any functional testing of candidate genes, both of these observations require further confirmation.

## Methods

### Mosquito sampling and wild *Plasmodium falciparum* infection

Mosquitoes were sampled as larvae using the standard dipping method as previously described [28]. In order to collect a random sample of essentially unrelated individuals, fewer than ten larvae were collected from any one larval site and a single collection comprised larvae from >50 larval sites. Collections were done in the Sudan Savanna region of Burkina Faso in the village of Goundry (12°30′N, 1°20′W), 30 km N of the capital city, Ouagadougou, across months of the malaria transmission season of 2007 and 2008 [29]. Larvae were raised in Centre National de Recherche et de Formation sur le Paludisme (CNRFP) insectaries in Ouagadougou to adulthood under standard insectary conditions [29]. Following emergence, 3-day old adults were challenged with wild *P. falciparum* by experimental infection. Feeding was done on an artificial membrane in a water-jacketed feeding device as described previously using gametocytaemic blood obtained from study participants [29]. Unfed mosquitoes were excluded from analysis and infection levels for fed mosquitoes were determined by counting midgut oocysts 7–8 days post infection. Genomic DNA was extracted from carcasses for SNP genotyping. Population sub-group composition was determined by published molecular diagnostic assays [28, 30]. Mosquitoes used for genetic association analysis came from five successful experimental infections on wild gametocytes using criteria defined previously [6, 28, 29, 31]: oocyst infection prevalence ≥30 % and oocyst intensity in at least one mosquito in the group of ≥10 oocysts. These criteria were applied to the mosquitoes raised together as a cohort and fed on the same blood, and identified the experiments with broad phenotype distributions. This infection-quality filter used for infection with wild gametocytes, which are inherently variable and noisy, assures that all analysed individuals came from an infection session with the power to distinguish between levels of susceptibility, free from confounding noise due to uninfective blood, technical failure or related factors. Low prevalence infections in the mosquitoes fed on gametocytaemic blood can potentially result from multiple causes, but regardless the cause, such groups are not informative for genetic analysis because of poor phenotype distribution. Mosquito genetics can be excluded as a cause, because the fed cohort is comprised of random unrelated individuals.

### SNP genotyping and genetic association

DNA samples from dissected mosquito carcasses were subjected to whole genome amplification (WGA) (Genomiphi, GE Health Sciences) using supplied protocols. Following WGA, DNA was ethanol precipitated, concentrations determined by the picogreen method [32], and 500 ng was used for SNP genotyping by the Illumina GoldenGate method [33] by the Boston Children’s Hospital Molecular Genetics Core Facility (IDDRC). The SNPs and their primer sequences are available in Additional file 1: Table S1. Data were analysed using BeadStudio software [33]. Genotypes of mosquitoes for the Pfin6/*para* haplotype were independently verified using the Sequenom MassArray method, with a set of seven diagnostic SNPs at the following genomic positions in the PEST reference genome: 2L.1272741 [T/C], 2L.1834476 [A/G], 2L.1970368 [C/T], 2L.2081228 [T/C], 2L.2430786 [C/T], 2L.2489023 [G/A], 2L.2489212 [T/C]. As these seven SNPs are in perfect linkage disequilibrium, they are all equally informative and thus comprise a sevenfold redundant assay for a single genotype feature, that is, the haplotype they are carried upon. Because the seven SNPs are all completely cross-informative for the haplotype, additional markers in this region would not increase resolution of the feature. For this, aliquots of the same DNA samples used for Illumina genotyping were genotyped using Sequenom at the University of Minnesota Genomic Center (UMGC). Sequenom genotypes were analysed for genetic association with malaria infection phenotype using the Haploview program [34].

### Gene silencing and functional assays

A colony of *An. coluzzii* mosquitoes (Fd03) was initiated with the eggs of ten mated, gravid females captured in village houses in Goundry, Burkina Faso. The colony is wild-type for *para* and is maintained using standard laboratory conditions at the Institut Pasteur. Double-stranded RNAs (dsRNA) were synthesized from PCR amplicons using the T7 Megascript Kit (Ambion) as described previously [35]. The sequences of the primers used for amplification of *ClipC9* dsRNA were:

T7_CLIPC9F:TAATACGACTCACTATAGGACCGTGCTGCAGAATGACTGC T7_CLIPC9R: TAATACGACTCACTATAGGTATCCCTAGTAGCACTAACCG.

The primers used for amplification of the *para*/VSC dsRNA were:

T7-4707F:TAATACGACTCACTATAGGAGGGCTATCCGGGAAATTGTGG.

T7-4707 R:TAATACGACTCACTATAGGTGAAGCGTCTGTTCCGCCTCC.

The underlined portion of the primer sequence is the T7 tag. For each targeted gene, 200 ng of dsRNA (but not more than 200 nl, depending on the concentration) were injected into the thorax of cold-anesthetized 1-day old *An. coluzzii* females using a microinjector (Nanoject II, Drummond). Validation of gene silencing efficiency was done by RT-PCR. In all cases, treatment with dsRNA led to decreases in target transcript abundance compared to dsGFP-injected controls (Additional file 2: Figure S1). The same numbers of mosquitoes were treated with control and experimental dsRNA in each replicate.

For gene silencing assays, mosquitoes were challenged with *P. falciparum* strain NF54 gametocytes, cultured using an automated tipper-table system implemented in the CEPIA mosquito infection facility of the Institut Pasteur, as previously described [35]. For experimental infection, 10 ml of medium containing mature gametocytes was centrifuged at 2000 rpm, and the cell pellet was resuspended in an equal volume of normal type AB human serum. The infected erythrocytes were added to fresh erythrocytes in AB human serum and transferred to a membrane feeder warmed to 37 °C. Mosquitoes were allowed to feed for 15 min. Unfed mosquitoes were removed, so that only fully engorged females were used for further analysis. Mosquitoes were maintained on 10 % sucrose solution supplemented with 0.05 % para-amino benzoic acid. At 7–8 days after the malaria infection challenge, all surviving mosquitoes were dissected. Thus, the dissected number, given as sample size (n) values below infection histograms, indicates survivorship, and control versus treatment values are compared as an estimate of gene-specific or overall mortality effects. At least two independent replicate infections were performed per condition. Replicates were analysed independently using the tests described below. If at least one replicate met the significance criterion of p ≤ 0.05, a third replicate was done.

### Analysis of infection phenotypes

For infections on either wild or cultured gametocytes, infection phenotype was determined at 7–8 days post blood meal. Mosquito midguts were dissected, stained with dibromofluorescein (Sigma Chemical) and the number of midgut oocysts determined by light microscopy. For mosquitoes fed on wild gametocytes, infection prevalence was measured (the fraction of mosquitoes with at least one oocyst), while for functional genomic tests using mosquitoes fed on cultured gametocytes, two infection phenotypes were measured, infection prevalence, and oocyst intensity, counts of oocysts in only those individuals with ≥1 oocyst. Oocyst intensity comparisons were not used for wild gametocyte infections because the sample size of mosquitoes with ≥1 oocyst did not provide sufficient statistical power for intensity.

For statistical analysis, comparisons of infection prevalence were made using the Chi Square test, and comparisons of oocyst intensity using the non-parametric Wilcoxon Mann–Whitney test. Replicates were analysed independently using the tests described above and p values from independent tests of significance were combined using the meta-analytical approach of Fisher [36], and this combined p value is reported here. The threshold for significance was defined as p = 0.01.

### Ethical considerations

For collection of blood from *P. falciparum* gametocyte carriers for experimental membrane feeder infection of mosquitoes, the study protocol was reviewed and approved by the institutional and national health ethical review board (Commission Nationale d’Ethique en Santé) of Burkina Faso (code No 2006-032). The study procedures, benefits and risks were explained to parents or legal guardians of children and their informed consent was obtained. Children of parents or guardians who had given consent were brought to CNRFP the day of the experiment for gametocyte carrier screening. All children were followed and symptomatic subjects were treated with the combination of artemether–lumefantrine (Coartem^®^) according to relevant regulations of the Burkina Faso Ministry of Health.

## Results

### Structure of the *para* gene locus

Wild mosquitoes were collected as larvae in Burkina Faso, were reared to adults in the insectary, and were challenged with wild *P. falciparum* parasites by membrane feeding on blood from gametocyte carriers, yielding 363 mosquitoes belonging to the *An. gambiae* species complex that were used in the study.

**Fig. 1:**
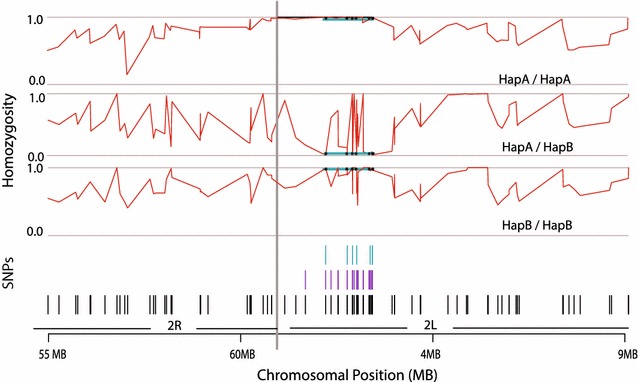
Fine structure of haplotypes in the *para* gene region measured as level of homozygosity. Mosquitoes were genotyped across ~15 Mb spanning the chromosome 2 centromere (*long vertical grey line*), first using a set of 105 SNPs (*black* SNP track) to detect the extent and approximate limits of the haplotype containing the *para* gene. Individuals were then grouped based on their genotype as defined by seven highly informative haplotype-tagging markers (*blue* SNP track), yielding three groups with haplotype status, HapA/HapA, HapA/HapB, HapB/HapB. Homozygosity was plotted for all individuals by genotype, depicted on the y axis as the fraction of homozygous individuals at each marker nucleotide position (for all individuals homozygous, y = 1; for all individuals heterozygous, y = 0). The region of homozygosity for the haplotype-tagging markers is indicated by a *horizontal blue line*, with the seven tag SNP positions marked by black stars within the *horizontal blue line*. The HapA allele carries the *kdr* mutation of the *para* gene

The mosquito DNAs were genotyped with 105 SNPs spanning a broad ~15 Mb region containing the *para* gene across the centromere of chromosome 2, between coordinates chromosome 2R:55 Mb to chromosome 2L:10 Mb (Additional file 1: Table S1; Fig. 1, bottom track of 105 SNPs shown as black vertical lines). Analysis of the individual mosquito genotypes reveals a region of elevated local homozygosity that defines the boundaries of two allelic haplotypes, termed HapA and HapB (Fig. 1). When mosquitoes were categorized by their HapA/HapB genotype, homozygotes are evident as regions of high linkage, with additional unlinked variation seen as dips in homozygosity (Fig. 1, homozygosity plot for each genotype). The core haplotype across both alleles is ~3 Mb in size (Fig. 1, blue horizontal lines superimposed on red homozygosity plot). This corresponds to the location and estimated 3.3 Mb size of the haplotype bearing the *kdr* allele of the *para* gene that was reported as an introgression from *An. gambiae* to *An. coluzzii* [24, 25].

**Fig. 2:**
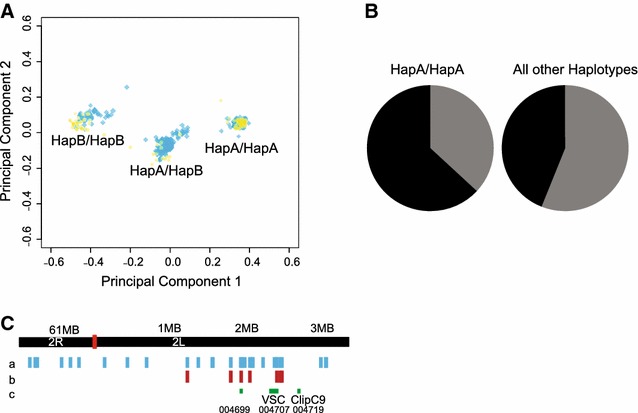
Population genetic association and analysis of haplotype locus. **A** Genetic markers in the *para* gene region of chromosome 2L clearly genotype haplotype alleles but do not distinguish between different population subgroups. Strong ancestral sharing of IBD genetic haplotypes across population subgroups indicates a shared common origin of the haplotype. *An. coluzzii* and *An. gambiae* (*yellow circles*), and Goundry form (*blue squares*), are shown on the PCA plot generated using the extended set of 26 haplotype-tagging SNPs (*purple* SNP track in Fig. 1). **B** Wild-collected individuals of the Goundry sub-group homozygous for HapA (n = 84) were significantly more susceptible to infection with natural *P. falciparum* than mosquitoes with any other haplotype (n = 180). *Pie chart* indicates mosquitoes infected with *P. falciparum* (*black*), and the uninfected mosquitoes (*grey*) after feeding together on blood from naturally infected donors. Unfed mosquitoes were removed from the experiment, thus all analysed mosquitoes ingested infectious parasites. **C** Pfin6 candidate genes indicated by Vectorbase AGAP gene ID, with positions of the functionally-tested genes *ClipC9* and *para*/VSC shown

The current sample set was comprised of three population groups of the *An. gambiae* species complex (*An. gambiae*, n = 46, *An. coluzzii*, n = 53, and Goundry form, n = 264). The Goundry form is a population sub-group within *An. gambiae* sensu lato with unknown taxonomic status or epidemiology for malaria transmission [28, 37, 38]. The haplotype structure of the *para* gene region in Goundry was not previously known. Of the 105 genotyped markers in this region, a sub-set of 26 SNPs span the haplotype (Fig. 1, middle track of SNPs shown as purple vertical lines) and were used to examine segregation of haplotype alleles across the three taxa. Consistent with observations that the *kdr* mutation introgressed from *An. gambiae* to *An. coluzzii* upon a haplotype [20, 21, 24, 25], the 26 SNPs in this genomic region did not distinguish between *An. gambiae*, *An. coluzzii* and the Goundry form (Fig. 2a). This result indicates that the *para*-bearing haplotype is identical by descent (IBD) across the three taxa, with strong ancestral sharing as well as the acquisition of additional mutations over time.

### Genetic association analysis for *Plasmodium falciparum* susceptibility

Tests for genetic association are done within panmictic population groups to avoid stratification artifacts [39]. Of the three population groups in the current study, *An. gambiae* have no power to test for association because the *kdr* allele is essentially fixed in *An. gambiae*, consistent with other reports [20, 21], and despite segregation of the *kdr* allele within *An. coluzzii*, the sample size was insufficient for an adequately powered statistical test. Thus, in the current study, only the Goundry group sample was appropriate for an association test of the *para*-bearing haplotype. As shown (Fig. 2A), a haplotype with a common evolutionary origin segregates in all three population groups. Thus, although the genetic background may differ among taxa, the same *para*-bearing haplotype is present in all of these taxa, and tests for association of the haplotype with *P. falciparum* infection levels were performed within the Goundry group.

A sub-set of seven completely correlated SNPs were chosen as haplotype-tagging markers for genetic association (Fig. 1, top track of SNPs shown as blue vertical lines). The seven SNPs mark ancestral differentiation, are in perfect linkage disequilibrium (LD) (thus are redundant tests for the same feature), and efficiently tag HapA and HapB alleles. Association of the *para*-bearing haplotype with susceptibility to *P. falciparum* was tested in the Goundry sub-group mosquitoes using the haplotype-tagging SNPs combined as a single redundant assay for the haplotype. Mosquitoes homozygous for haplotype allele HapA were more susceptible to infection with *P. falciparum* than heterozygotes or HapB homozygotes (p = 0.0293 for oocyst infection prevalence; Fig. 2B). The locus was named *Plasmodium falciparum infection locus 6* (Pfin6) following naming convention [31].

### Functional dissection of the Pfin6 locus

The haplotype that carries the mapped Pfin6 locus as well as the *para* gene is ~3.3 Mb in length [24, 25] and contains 65 predicted genes (Fig. 2C; Additional file 3: Table S2). Because the haplotype contains long blocks of SNPs in perfect linkage, resolution by recombinational fine-mapping is not expected to be productive. Fine-mapping would be further complicated by the centromeric location of the mapped locus, as centromeres typically have lower than average recombination rates. Instead, in the present study a candidate gene approach is employed, dissecting the locus by functional testing of candidates ascertained by predicted gene function and other evidence. It is important to note that candidate gene assays can provide suggestive evidence, but do not constitute proof of genetic causation.

Of the genes in the locus (AGAP004677-AGAP004742), two are potentially involved in host-defense processes: AGAP004719 (2L:2.71 Mb), with strong prediction of immune function, identified as *ClipC9*, a clip-domain serine protease homologous with the *Drosophila* extracellular serine protease Persephone (CG6367), which mediates activation of the Toll signalling pathway in response to fungal infection [40]; and AGAP004699 (2L:1.97 Mb), weakly predicted as potentially immune, which codes for a serine/threonine-protein kinase with a *Drosophila* orthologue involved in haemocyte differentiation (Raf homologue serine/threonine-protein kinase phl, CG2845, [41]. The locus also contains AGAP004707 (2L:2.35 Mb), the *para* gene in which the *kdr* mutant is associated with response to pyrethroid insecticides.

The following lines of evidence prioritized the functional testing of candidates *ClipC9*/AGAP004719 and *para*/AGAP004707 for their effect on the outcome of *P. falciparum* infection, using RNAi-mediated gene silencing assays. *ClipC9* is predicted to be catalytically active as a serine protease, bearing all cysteine residues involved in disulfide bonds, as well as the conserved residues of the catalytic triad. Expression of *ClipC9* was upregulated following infection with Gram-negative bacteria and down-regulated following infection with Gram-positive bacteria [42, 43], but was unchanged following infection with *Plasmodium* [43]. The *para* gene would not be selected by the same criteria as a candidate for functional testing, because it does not display any known or predicted immune function, but it was chosen on an ad hoc basis because of reports of association of *kdr* with infection [26, 27]. The *para* gene influence upon any phenotype has not been functionally analysed by gene silencing, according to published reports.

**Fig. 3:**
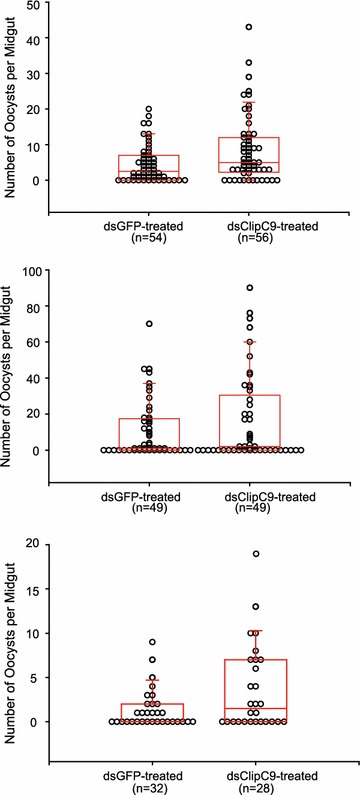
Candidate gene ClipC9 within the Pfin6 locus protects mosquitoes against infection with *Plasmodium falciparum*. Expression of the ClipC9 (AGAP004719) gene was silenced by dsRNA injection, mosquitoes were challenged with cultured *P. falciparum* gametocytes, and infection outcomes were compared with control mosquitoes treated with dsGFP. In all three replicate experiments, the dsClipC9-treated mosquitoes permitted development of significantly higher numbers of midgut oocysts as compared to controls (combined p value, 5.16 × 10^−4^; three replicate experiments are shown with *box plots*, p values from individual experiments were combined using the Fisher Method). Thus, the activity of ClipC9 influences *P. falciparum* oocyst infection intensity in *A. gambiae*. There was no difference in oocyst infection prevalence between ClipC9-silenced mosquitoes and GFP controls (p = 0.78)

Mosquitoes from an *An. coluzzii* colony recently initiated from the Burkina Faso study site were injected with double-stranded RNA (dsRNA) targeting *ClipC9*, *para*, or control GFP. Four days after dsRNA treatment, decreased transcript levels were verified (Additional file 2: Figure S1), mosquitoes were fed on cultured *P. falciparum* gametocytes, and 7–8 days later midgut oocysts were counted. Infection intensity (numbers of oocysts in mosquitoes with ≥1 oocyst) was consistently higher in the ds*ClipC9*-treated individuals as compared to controls (Fig. 3; p = 6.23 × 10^−4^; p values from three replicate experiments combined using the Fisher Method; individual p values 0.005, 0.089, 0.017). There was no effect of ds*ClipC9* treatment upon infection prevalence (the proportion of mosquitoes with ≥1 oocyst, p = 0.78; individual p values 0.658, 0.678, 0.450).

**Fig. 4:**
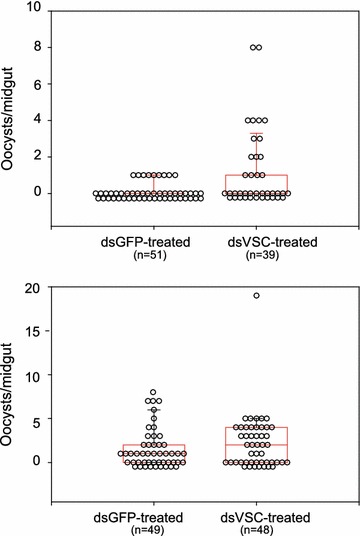
RNA-mediated gene silencing of the *para* gene does not influence *Plasmodium falciparum* oocyst infection levels. The results of two replicate experimental infections are shown. Dot plots indicate the number of midgut oocysts in all individual mosquitoes. Neither infection prevalence (p = 1.0) nor infection intensity (p = 0.10) were statistically significant between mosquitoes silenced for *para* gene transcript (dsVSC) and control mosquitoes treated with dsGFP

For the *para* voltage-gated sodium channel (VSC) gene, there was no statistically significant difference for either infection prevalence or intensity between ds*VSC*-treated as compared to control mosquitoes (infection prevalence p = 1.0, infection intensity p = 0.10, Fig. 4). Thus, under these conditions, abolishing *para* gene transcriptional activity does not appear to influence parasite development, although the effect of transcript silencing on protein levels is not known.

Testing gene function by silencing, as shown here, is distinct from testing allele function by genetic association, as shown above. Gene silencing mediated by a ~500 bp double-stranded RNA targeting the gene is immune to sequence differences greater than the *kdr* SNP variant between *para* alleles [44]. The colony used for the functional test was fixed for wild-type *para*, and transcript silencing is efficient (Additional file 2: Figure S1). Thus, *para* gene transcripts are abolished by silencing, regardless of the genetic variants they carry.

The effect of gene silencing on mosquito mortality was monitored by comparison of control dsGFP and experimental dsRNA treatments. The same numbers of control and experimental mosquitoes were injected with dsRNA per replicate, and all surviving mosquitoes were dissected, thus the sample sizes (n values shown on Figs. 3, 4) indicate overall survival per treatment. There was no evidence of a consistent difference in survival between mosquitoes treated with ds*ClipC9* as compared to ds*GFP* controls (Fig. 3), or between ds*VSC*-treated as compared to ds*GFP* controls (Fig. 4). Thus, it appears that neither *ClipC9* nor *para* gene activity is biologically essential for mosquito viability, although minor effects on survival cannot be ruled out.

## Discussion

### The Pfin6 malaria infection locus and *kdr* are carried on the same haplotype

Results presented here show that a ~3 Mb haplotype is shared by introgression as an identical by descent (IBD) feature among three otherwise reproductively isolated groups of the *An. gambiae* species complex. Two of the three groups, *An. gambiae* and *An. coluzzii*, display overall equivalent susceptibility to wild *P. falciparum* [29, 45–48], while the Goundry form is more susceptible to experimental infection with wild parasites [28]. However, because the haplotype is a shared feature with common evolutionary origin among the three population groups, it is unlikely to explain this phenotypic difference between the groups.

The unit of introgression of the *kdr* allele was not simply the *kdr* variant nucleotide, but rather an extended haplotype bearing *para* and >60 other neighbouring genes. The ~3 Mb size of the introgressed haplotype reported here is consistent with other reports [24, 25]. It has been suggested that exposure to insecticides used in agriculture or on bed nets could be driving the spread of *kdr*, based on the temporal correlation of the two events [23, 49, 50]. The finding that the pathogen-resistance locus, Pfin6, maps to the same haplotype suggests a potential complementary explanation for the adaptive introgression of this haplotype. Components of the immune systems of *An. coluzzii* and *An. gambiae* mosquitoes are under distinct selective pressure that has strongly altered the alleles and diversity at particular immune loci unrelated to Pfin6 [37, 51, 52]. In those cases, it is hypothesized that exposure to distinct pathogen profiles in the different ecological niches of *An. coluzzii* and *An. gambiae* mosquitoes could be the source of this strong recent selection. The presence of a functional immunity locus upon this haplotype might explain at least part of the introgression pressure based on differential immunity to environmental microbes.

### Functional genomic analysis of the haplotype carrying Pfin6 and *para*

Based on prediction of potential immune function one high-priority candidate gene was identified within the Pfin6 locus, the clip domain serine protease, *ClipC9*. This does not rule out that a different or multiple genes could actually underlie the *Plasmodium* susceptibility phenotype of Pfin6 detected in nature. *ClipC9* was functionally confirmed, by gene silencing, as a gene that influences mosquito infection levels with *P. falciparum*. Functional assays of candidate genes can demonstrate that a gene influences a similar phenotype to a genetically mapped locus, and thus could be a plausible genetic candidate. However, functional assays are not proof of genetic causation. Subsequent work will be required to establish whether naturally-occurring genetic variation in *ClipC9* underlies the genetic effect of the Pfin6 locus in wild mosquitoes.

### Pfin6 linkage with *kdr* and malaria transmission

The observed linkage reported here between *P. falciparum* susceptibility and insecticide resistance, and observations in other recent studies using different methodological approaches show the same direction of association [26, 27]. The functional analysis of *para* suggests that *para* itself is not responsible for the malaria susceptibility phenotype, although an effect in nature cannot be ruled out. The influence of transcript loss on protein abundance could not be measured because of the lack of existing antibodies against this highly hydrophobic protein. Protein abundance is often safely ignored in functional genomic experiments that produce a detectable phenotype, although in the case of *para* the absence of a transcript depletion phenotype for either infection or mortality means that the unknown protein level remains a caveat.

In principle, wild *kdr*-bearing mosquitoes could contribute disproportionately to malaria transmission if they live longer than wild-type mosquitoes, although the comparative lifespan of *kdr* mosquitoes in field populations is not known. Here, mosquitoes were not exposed to insecticide treatment, and all individuals were offered a single blood meal and dissected 7–8 days afterwards, so any *kdr*-based differences in longevity, if they exist, do not influence the results.

Loss of *para* transcript by gene silencing did not result in higher mosquito mortality. This viability result is consistent with observations from the para orthologue in *Drosophila* (FlyBase ID FBgn0264255), which harbours many described mutations, some lethal but many viable [53], often displaying temperature-sensitive behavioural defects such as excitability that have been used as models for neurological disorders [54, 55]. Based on the finding that mosquitoes depleted for *para* transcript are viable, it would be interesting to assay *para*-silenced mosquitoes for influence on other behavioral phenotypes that could be related to insecticide response.

## Conclusion

It is a worrisome observation that mosquitoes genetically resistant to the pyrethroid insecticides commonly used on insecticide-treated bed nets may also be more susceptible to *P. falciparum*, and thus could potentially be more efficient malaria vectors. This effect might partially compensate for lower rates of human contact with mosquitoes after bed net distributions, and could be related to several puzzling reports of unexpected levels of malaria despite vector-control interventions [56–58].

## Additional files

10.1186/s12936-015-0924-8 Catalogue of polymorphic SNPs used in the study.
10.1186/s12936-015-0924-8 Verification of gene silencing of the ClipC9 and para genes by treatment of mosquitoes with specific dsRNAs. Transcript detected by RT-PCR is indicated to the left of the gel image for ClipC9 and to the right of the gel for para. Detection of mRNA for ribosomal protein S7 (rpS7) was used as an internal reference.
10.1186/s12936-015-0924-8 Annotated coding sequences within the haplotype carrying the Pfin 6 locus and para gene.
