# Supplementary material for: The kdr-bearing haplotype and susceptibility to Plasmodium falciparum in Anopheles gambiae: genetic correlation and functional testing
Source: Malar J. 2015 Oct 6;14:391. doi: 10.1186/s12936-015-0924-8 (PMC4596459; doi:10.1186/s12936-015-0924-8)
Supplement: Supplementary file 2 — 10.1186/s12936-015-0924-8 Verification of gene silencing of the ClipC9 and para genes by treatment of mosquitoes with specific dsRNAs. Transcript detected by RT-PCR is indicated to the left of the gel image for ClipC9 and to the right of the gel for para. Detection of mRNA for ribosomal protein S7 (rpS7) was used as an internal reference. [file 12936_2015_924_MOESM2_ESM.pdf]

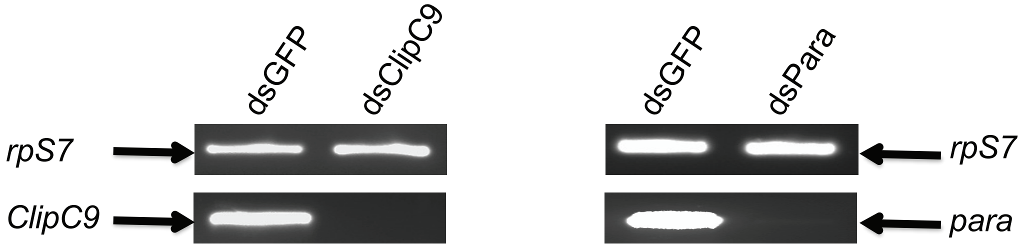

Figure S1. Verification of gene silencing of the ClipC9 and para genes by treatment of mosquitoes with specific dsRNAs. Transcript detected by RT-PCR is indicated to the left of the gel image for ClipC9 and to the right of the gel for para. Detection of mRNA for ribosomal protein S7 (rpS7) was used as an internal reference.
