# Supplementary material for: The kdr-bearing haplotype and susceptibility to Plasmodium falciparum in Anopheles gambiae: genetic correlation and functional testing
Source: Malar J. 2015 Oct 6;14:391. doi: 10.1186/s12936-015-0924-8 (PMC4596459; doi:10.1186/s12936-015-0924-8)
Supplement: Supplementary file 3 — 10.1186/s12936-015-0924-8 Annotated coding sequences within the haplotype carrying the Pfin 6 locus and para gene. [file 12936_2015_924_MOESM3_ESM.docx]

| **Additional file 3: Table S2. Annotated coding sequences within the haplotype carrying the Pfin 6 locus and para gene.** | | | | | |
| --- | --- | --- | --- | --- | --- |
|  |  |  |  |  |  |
| **Gene stable ID** | **Gene start (bp)** | **Gene end (bp)** | **Gene name** | **Chromosome** | **Gene description** |
| AGAP004677 | 157348 | 186936 |  | 2L | methylenetetrahydrofolate dehydrogenase(NAD+) / 5,10-methenyltetrahydrofolate |
| AGAP004678 | 203779 | 205293 |  | 2L |  |
| AGAP004679 | 207894 | 210460 |  | 2L | folylpolyglutamate synthase |
| AGAP004680 | 271285 | 271815 |  | 2L |  |
| AGAP004681 | 358329 | 359280 |  | 2L |  |
| AGAP004682 | 433503 | 461627 |  | 2L | U4/U6 small nuclear ribonucleoprotein PRP3 |
| AGAP004683 | 485698 | 488369 |  | 2L |  |
| AGAP004684 | 493039 | 493543 |  | 2L | rRNA-processing protein CGR1 |
| AGAP004685 | 493579 | 497632 |  | 2L | DNA replication ATP-dependent helicase Dna2 |
| AGAP004686 | 742004 | 747057 |  | 2L |  |
| AGAP004687 | 819113 | 819301 |  | 2L |  |
| AGAP004688 | 1018044 | 1019196 |  | 2L |  |
| AGAP004689 | 1052036 | 1057434 |  | 2L |  |
| AGAP004690 | 1133037 | 1133751 | CPF3 | 2L | cuticular protein 3 from fifty-one aa family |
| AGAP004691 | 1272330 | 1301008 |  | 2L | LIM domain-binding protein 1 |
| AGAP004692 | 1411011 | 1441380 |  | 2L |  |
| AGAP004693 | 1527686 | 1533479 |  | 2L | nuclear receptor subfamily 6 group A |
| AGAP004694 | 1595176 | 1602296 |  | 2L |  |
| AGAP004695 | 1834186 | 1835605 |  | 2L | ESCRT-I complex subunit MVB12 |
| AGAP004696 | 1926520 | 1965505 |  | 2L | Homeobox protein extradenticle |
| AGAP004697 | 1929925 | 1929996 | tRNA-Pro | 2L | tRNA-Pro for anticodon AGG |
| AGAP004698 | 1969023 | 1971192 |  | 2L | pre-mRNA-splicing factor 38B |
| AGAP004699 | 1973601 | 1976357 |  | 2L | RAF proto-oncogene serine/threonine-protein kinase |
| AGAP004700 | 2013634 | 2015127 |  | 2L |  |
| AGAP004701 | 2074972 | 2120041 |  | 2L |  |
| AGAP004702 | 2163142 | 2164726 |  | 2L |  |
| AGAP004703 | 2248667 | 2253660 |  | 2L | DNA-directed RNA polymerase III subunit RPC1 |
| AGAP004704 | 2253748 | 2255495 |  | 2L | COMPASS component SPP1 |
| AGAP004706 | 2305657 | 2306118 |  | 2L |  |
| AGAP004707 | 2358158 | 2431617 | para | 2L | voltage-gated sodium channel |
| AGAP004708 | 2471997 | 2474401 |  | 2L | arginyl-tRNA synthetase |
| AGAP004709 | 2482553 | 2483310 |  | 2L | large subunit ribosomal protein L18 |
| AGAP004710 | 2483226 | 2483631 |  | 2L | ubiquinol-cytochrome c reductase subunit 9 |
| AGAP004711 | 2487770 | 2489611 |  | 2L | ATP-dependent RNA helicase DDX41 |
| AGAP004712 | 2506572 | 2507341 |  | 2L |  |
| AGAP004713 | 2559360 | 2559808 |  | 2L |  |
| AGAP004714 | 2573498 | 2574460 |  | 2L |  |
| AGAP004715 | 2615657 | 2620722 |  | 2L | Pyruvate dehydrogenase phosphatase regulatory subunit, mitochondrial |
| AGAP004716 | 2624121 | 2627818 | Gr57 | 2L | gustatory receptor |
| AGAP004717 | 2643650 | 2679635 |  | 2L |  |
| AGAP004718 | 2687938 | 2714108 |  | 2L |  |
| AGAP004719 | 2714472 | 2719933 | CLIPC9 | 2L | Clip-Domain Serine Protease |
| AGAP004720 | 2727394 | 2742060 |  | 2L |  |
| AGAP004721 | 2749855 | 2752163 |  | 2L | N-acetylglucosamine-6-sulfatase |
| AGAP004722 | 2752351 | 2753362 |  | 2L | Co-chaperone protein HscB, mitochondrial |
| AGAP004723 | 2756472 | 2760324 |  | 2L | Chromobox protein homolog 1 |
| AGAP004724 | 2775211 | 2796481 |  | 2L | Intraflagellar transport 74 homolog |
| AGAP004725 | 2797664 | 2801444 |  | 2L | Eukaryotic translation initiation factor 3 subunit C |
| AGAP004726 | 2804809 | 2807762 |  | 2L | Uncharacterized protein CG3556 |
| AGAP004727 | 2812213 | 2814510 | Gr25 | 2L | gustatory receptor |
| AGAP004728 | 2824043 | 2831367 |  | 2L |  |
| AGAP004729 | 2844659 | 2847575 |  | 2L |  |
| AGAP004730 | 2865742 | 2866766 |  | 2L | Phospholipase A2, venom |
| AGAP004731 | 2867519 | 2886486 |  | 2L | secretory phospholipase A2 |
| AGAP004732 | 2898242 | 2906165 |  | 2L |  |
| AGAP004733 | 2917655 | 2919864 |  | 2L |  |
| AGAP004734 | 2920492 | 2930688 |  | 2L | PAX-interacting protein 1 |
| AGAP004735 | 2966120 | 2967485 |  | 2L |  |
| AGAP004736 | 2968484 | 2969976 |  | 2L | Mitochondrial GTPase 1 homolog |
| AGAP004737 | 2970597 | 2972774 |  | 2L | Rhomboid-4, isoform B |
| AGAP004738 | 2972617 | 2974653 |  | 2L | IK cytokine |
| AGAP004739 | 2976111 | 2977915 |  | 2L | H/ACA ribonucleoprotein complex subunit 4 |
| AGAP004740 | 2982984 | 2983998 |  | 2L | Serine collagenase 1 precursor |
| AGAP004741 | 2997029 | 2998143 |  | 2L | serine proteinase |
| AGAP004742 | 2998916 | 3008084 |  | 2L | pyruvate carboxylase |
|  |  |  |  |  |  |
